# Application of imaging modalities for endovascular thrombectomy of large core infarcts in clinical practice

**DOI:** 10.3389/fneur.2024.1272890

**Published:** 2024-04-11

**Authors:** Hai Zeng, Qingfeng Zhu

**Affiliations:** Neurosurgery, Second Hospital of Shanxi Medical University, Taiyuan, China

**Keywords:** large infarct core volume, endovascular thrombectomy, ASPECTS, imaging, non-contrast CT

## Abstract

Four randomized controlled trials of large infarct core volume (LICV) included three imaging modalities: non-contrast CT (NCCT)-Alberta Stroke Program Early CT Score (ASPECTS), diffusion-weighted imaging (DWI)-ASPECTS, and NCCT-ASPECTS combined with CTP (CT perfusion). However, there is no clear consensus on the optimal imaging modality for endovascular thrombectomy (EVT) trials of large core infarcts. The variety and complexity of imaging modalities make it difficult to apply them in clinical practice. By familiarizing ourselves with these imaging modalities, we can better apply them in the clinic and correctly screen patients with large core infarcts in the anterior circulation who can benefit from EVT therapy.

## 1 Introduction

Large core ischemic stroke accounts for approximately 20% of acute ischemic stroke-large vessel occlusions (AIS-LVOs) (1). Several studies have shown that endovascular thrombectomy (EVT) is feasible and safe for large infarct core volumes (LICVs) (1–4). ASPECTS is a widely accepted tool used for assessing infarct volume, and its applicability has been recognized by numerous randomized controlled trials (RCTs) (5–10). Although the current study confirms that EVT is an effective treatment for conditions characterized as ASPECTS 6–10, early RCTs on EVT excluded patients with ASPECTS 0–5 or infarct volumes ≥70 mL or ≥ 50 mL. Furthermore, it has been suggested that such patients would not benefit from EVT, and they were classified as having LICV. Thus, the efficacy and safety of EVT therapy in patients with LICV remain unclear. However, with the publication of the RESCUE-Japan (11), ANGEL-ASPECT (12), SELECT2 trials (13), and the TENTION trial (14), which confirmed the superiority of the EVT treatment over pharmacologic therapy, high-level evidence-based medical data have emerged supporting the EVT treatment for LICV. Although the results of these four trials are positive, their imaging methods differ, presenting complex and diverse characteristics, making it difficult for clinical applications. Choosing the appropriate imaging mode is crucial in clinical applications. This article comments on the imaging patterns of large core infarctions.

## 2 How to select patients who benefit from EVT treatment in acute large core infarcts?

1. The RESCUE-Japan LIMIT trial imaging criteria (11): patients who presented within 6 h after the onset of symptoms and utilized diffusion-weighted imaging (DWI), or DWI/fluid-attenuated inversion recovery signal (6–24 h) to evaluate cases classified as ASPECTS 3–5 (28.6% of the patients were selected based on this method). Occluded vessels were the internal carotid artery (ICA) or middle cerebral artery (MCA)-M1 or M2 (see Table 1).
2. The ANGEL-ASPECT trial imaging criteria (12): patients meeting any of the following criteria were included: ASPECTS 3–5, regardless of infarct volume; ASPECTS >5 (6–24 h) with an infarct core volume of 70–100 mL (defined as an area with a relative cerebral blood flow (rCBF) of <30% or ADC of <620 × 10–6 mm^2^ on CTP); and ASPECTS of <3 with an infarct core volume of 70–100 mL. Occluded vessels were ICA, MCA-M1, or M2.
3. The SELECT2 trial imaging criteria (13): patients who presented within 24 h from symptoms onset and included cases with an NCCT-ASPECTS of 3–5 or ischemic core volume of ≥50 mL (defined as an area with a rCBF of <30% or ADC of <620 × 10–6 mm^2^ on CTP). Occluded vessels were either MCA-M1 or M2.
4. The TENSION trial imaging criteria (14): patients with NCCT-ASPECTS of 3–5 within 12 h after onset with either ICA or MCA-M1 or M2 occlusion.

**Table 1:** Trials inclusion criteria.

Trials	RESCUE-Japan LIMIT	ANGEL-ASPECT	TENSION	SELECT 2
Imaging inclusion Criteria	DWI-ASPECTS 3–5	(1). ASPECTS 3–5 (2). ASPECTS >5 (>6 h) and core 70–100 mL (3). ASPECTS <3 and core 70–100 cc	NCCT or DWI:ASPECTS 3–5	(1). ASPECTS≥6 andcore50mL (2). ASPECTS 3–5 and core>50 mL (3). ASPECT 3–5 an core<50 mL
NIHSS	≥6	6–30	<26	≥6
Age	>18	18–80	>18	18–85
Time	<24 h	<24 h	<12 h	<24 h

All the above four trials showed that EVT treatment was superior to drug treatment. We can refer to the imaging criteria used in any of these tests to screen for patients with a large core pre-circulation infarction. They showed that patients with NCCT or MRI-ASPECTS of 3–5 could benefit from EVT within 24 h. In addition, the ANGEL-ASPECT trial and SELECT2 trial also showed that patients with infarct volumes ≥50 mL or 70–100 mL could benefit from EVT within 24 h. Although the risk of intracranial hemorrhage is increased compared to drug therapy, the overall benefits of EVT treatment outweigh this risk.

## 3 Discussion

Four trials included three imaging modalities: DWI-ASPECTS, NCCT-ASPECTS, and NCCT-ASPECTS combined with CTP. It is important to gain a sufficient understanding of these modalities to facilitate their clinical application.

The cutoff range of ASPECTS 0–5 for large core cerebral infarcts is widely agreed upon and is not controversial (10, 15). The ASPECTS is often evaluated utilizing either DWI-MRI or NCCT, both of which provide highly specific indicators from a pathophysiological standpoint (16, 17). The trials confirmed the efficacy of ASPECTS 3–5 in EVT; however, they did not demonstrate the efficacy of ASPECTS 0–2.

NCCT and DWI-MRI are often considered simple, fast, and widely used imaging modalities in most hospitals, and they are extensively employed in clinical practice. DWI-ASPECTS is more sensitive than NCCT-ASPECTS for early acute ischemic changes. However, the reversible changes in DWI may lead to false-positive test results and prolong the time for vessel recanalization due to longer imaging time, which is unfavorable for patient prognosis. These are the limitations of DWI. DWI high signal does not necessarily represent irreversibly infarcted tissue, because most DWI-detected injuries are reversible. Yoo et al. (18) reported that complete reperfusion and shorter imaging-to-reperfusion time after EVT treatment were independently associated with DWI reversal. Moreover, other studies have shown a major difference in the rate of DWI lesion reversal, ranging from 0 to 83%, with a mean reversal rate of 24% (19). This suggests that the favorable prognosis obtained after revascularization in patients with LICV enrolled using the DWI-ASPECTS imaging modality may be partly due to reversible changes in the DWI lesion. Therefore, a greater proportion of reversals in DWI could likely lead to a positive trial outcome. In addition, prolonged duration of DWI examination is detrimental to EVT treatment for LICV patients. The RESCUE-Japan trial sub-analysis indicates that early reperfusion was associated with a favorable prognosis in patients with acute large vessel occlusion and a large ischemic area (20).

NCCT has a lower reversal ratio, which can more accurately describe the actual infarction situation. Moreover, NCCT is faster than DWI and is widely used in Western countries. They rely mainly on the visual assessment of the early ischemic signs, primarily through NCCT, supplemented by CT angiography, to determine the location of vascular occlusion for the management of patients with acute stroke in clinical practice (14). However, NCCT-ASPECTS is 1 point higher than DWI-ASPECTS in the assessment (21). Due to the common occurrence of such differences in clinical practice, the TENTION trial (14) primarily used NCCT-ASPECTS as the main imaging modality. However, a limitation is that inconsistencies in NCCT-ASPECTS scoring between evaluators (22–24), especially at critical thresholds, can lead to incorrect patient allocation to the EVT and medical group, thereby compromising the reliability of the trial conclusions.

In addition to using ASPECTS, the ANGEL-ASPECT and SELECT2 trials also utilize rapid processing of perfusion and diffusion (RAPID) to calculate the infarct volume in the CBF region under CTP. CTP has three main advantages. First, it can accurately measure the volume of the ischemic area and compensate for the differences between evaluators when using NCCT-ASPECTS alone (2), as it can correctly classify large core infarctions. Second, it can include more patients with large core strokes to determine the lower limit of benefit in EVT treatment. Third, CTP helps identify the penumbra/infarct area and its significance in large core strokes. However, this method has been highly controversial. As Jadhav et al. highlighted in a previous review, a single snapshot of CBF lacks accuracy (25), and several studies have shown that measuring CTP in an early window may overestimate the true infarct volume, making it challenging to ensure accuracy (24, 26–29). Thus, ANGEL-ASPECT and SELECT2 trails only use CTP measurements under specific conditions to improve the accuracy of CTP. CTP measurements in ANGEL-ASPECT were used only in two situations: first, in patients with ASPECTS >5 in a late window (6–24 h) to ensure CTP accuracy and to offset the differences between NCCT-ASPECTS assessors that may lead to the misallocation of patients; and second, in patients with ASPECTS 0–2 (0–24 h) and a core infarct volume of 70–100 mL, the reason for using EVT is because previous studies have not provided clear evidence from EVT in patients with ASPECTS 0–2. This subgroup aims to analyze and determine if patients with ASPECTS 0–2 can benefit from EVT treatment and establish the lower limit of benefit. SELECT2 used CTP-estimated core volumes in both the early (0–2 h only using CBF <20% as a strategy to improve accuracy) as well as late windows. Although studies have indicated that the infarct volume under CTP can replace ASPECTS on NCCT or DWI-MRI (30, 31), the high cost of this imaging modality limits its practical use. Meanwhile, the trial centers using CTP were all located in areas with developed medical resources, making it difficult to popularize them in areas that lack medical resources.

CTP can identify penumbra/core mismatches. Multiple studies (32–35) have shown that patients with small to medium-sized ischemic strokes with penumbra/core mismatch can significantly benefit from EVT treatment, but it is currently unclear whether it is effective in large core infarction patients. A meta-analysis showed (34) that in RCTs that included patients undergoing CTP, many patients had a mismatch in baseline imaging. One study (2) also found that approximately 50% of patients had a large core infarct mismatch within 6 h of the last examination, and only mismatched patients appeared to benefit from EVT. However, it has also been shown that the penumbra has a low incidence in cases with a low ASPECTS. The incidence of mismatch decreased with lower ASPECTS (6.4%/1 score) (36); CTP was predictive of functional prognosis after undergoing EVT but did not accurately identify patients who failed to benefit from EVT. Based on the low occurrence rate of ASPECTS 0–5 mismatch, including penumbra/core mismatch as an inclusion criterion may not have much benefit and may even pose a risk of delaying vascular reperfusion. The four trials (11–14) did not include penumbra/core mismatch among the inclusion criteria; it may be possible to go through a pooled analysis to clarify the role of penumbra/infarct mismatch in patients with EVT. Some scholars (2) have suggested clinical/infarct mismatch as an alternative to penumbra/core mismatch as an inclusion criterion, such as the ongoing LASTE trial (NCT03805308).

Collateral status is also a good prognostic indicator (37). Among patients with ASPECTS <6, those with good collaterals have better clinical outcomes with EVT compared to those without good collaterals (38). Low ASPECTS is associated with lower infarct volume but not with higher penumbra volume (38), indicating that collateral circulation plays a major role in preventing early tissue loss and suggesting that the role of the penumbra may be limited in large core infarcts. Although collateral status was not included as part of the inclusion criteria in the trials, it can be used to determine the role of collateral status in EVT patients with ASPECTS 0–5 using subgroup analysis.

## 4 Conclusion

Every imaging modality has its limitations. The current practice should select the appropriate imaging approach based on the local medical resource availability to screen out large core ischemic stroke patients who could benefit from EVT (for example, using NCCT or MRI in areas with limited medical resources, using multiple imaging modes in those with advanced medical resources). Delaying patients’ EVT treatment should not occur due to long imaging examinations or the absence of CTP examinations.

## Author contributions

HZ: Writing – original draft, Validation. QZ: Writing – review & editing, Supervision, Funding acquisition.
